# Biographical

**Published:** 1895-03

**Authors:** 


### Biographical.

William O. Kulp was a native of Ohio, having been born at Wadsworth, O., Sept. 19, 1836. He had, therefore, entered upon his fifty-ninth year at the time of his death. He was remotely of German ancestry, his family having come from Germany about two hundred years ago. In the public schools and under private tutelage he received a good education. At the age of twenty he commenced the study of medicine, and three years later came to Muscatine, Ia. Trouble with his teeth and the difficulty he experienced in its alleviation turned his attention to dentistry, and he decided to enter that line of work. In Muscatine he became the junior partner of H. G. Hall, a prominent dentist of that place. It was in the school of experience that he laid the foundation for that profound knowledge of operative dentistry that made him afterward one of the best-known members of his profession in this country. After a couple of years Dr. Hall retired, and Dr. Kulp, after perfecting himself in a course of study at the Missouri Dental College, from which he graduated in 1866, practiced alone in Muscatine for the succeeding decade, coming to Davenport in 1871. Here his successful business career is a part of the history of the city with which everyone is familiar. He was first associated with Dr. White, practiced long by himself, and in 1893 admitted his son, Dr. J. R. Kulp, to a partnership, under which their business has since been successfully conducted.

It was through the efforts of Dr. Kulp that the Iowa Dental Association was organized in 1863. Since 1864 he has been a member of the American Dental Association. In 1887 he was called to the chair of operative dentistry in the Missouri Dental College, but illness in his family caused him to resign the position and return to Muscatine after a year. Upon the organization of the dental department of the Iowa State University, some fifteen years ago, he was chosen as the lecturer upon operative dentistry and dental therapeutics, and he has discharged the duties of the office since that time in a manner that has won for him the admiration and unqualified approval of the board of regents and of all who came under the spell of his lectures. He will not be easily replaced upon the faculty of the university.

He was one of the principal supporters of the movement which resulted in the State regulation of the practice of dentistry in Iowa, and was one of the vice-presidents of the World's Columbian Dental Congress, one of the important meetings of 1893. The congress was attended by representatives of forty-five countries, and the paper Dr. Kulp read before that body received much flattering comment.

In various fields has the progressive spirit of the deceased made itself felt. He was a member of the board of directors and was treasurer of the Pan-American Transport Company, a steamship concern in the possession of valuable franchises and reaching out after South American commerce and mail contracts.

He was also secretary of the Western Land and Live Stock Company, which owns 100,000 acres of land and 30,000 head of cattle.

As a member of the executive committee of the movement for a deep water harbor on the Texas Gulf coast, he presented the claims of that project to the appropriate congressional committee, and before his death had the pleasure of seeing his desires in this connection in a fair way for realization.

In addition to these he was interested and active in many other important and public interests, in which whatever he did, was characterised by faithfulness and efficiency.

He was ever the friend and advocate of the right, and uncompromising against the wrong. His friendships were usually strong and enduring.

He was a man of strong intellectual force, and of a high moral and religious nature.

His loss is a serious one, not only to his family and to the community in which he lived, but to the profession of his state, and indeed to the profession of the entire country.

May his memory ever be cherished as a model and an example worthy to be followed by young and old, and thus may his name be honored henceforth.

